# The Demobilisation of Temporary Officers in the R.A.M.C.

**Published:** 1919-08-16

**Authors:** 


### THE DEMOBILISATION OF TEMPORARY OFFICERS IN THE R.A.M.C.

Some six months or so back much indignation was expressed, both in the medical profession and outside it, when the War Office was forced to confess (in answer to a question in the House of Commons) that the proportion of R.A.M.C. officers still in the B.E.F. to the total personnel of that force was actually higher than it had been at the date of the Armistice three months or more previously. The scandal was so glaring that Mr. Churchill could but admit it, and stated that he had issued urgent orders for the rectification of the absurdity. These orders were, for a time at any rate, complied with; but it was never officially stated who was responsible for the *bêtise*, or whether any effort had been made to fix such responsibility. The upshot was that in another month or two—say by the beginning of May—there were few married officers still undemobilised who had been in practice before joining the Army; this class, quite rightly, had priority of the unmarried or those not previously in practice.

Since that time complaints have again been gathering force about the slowness of demobilisation of those doctors who are still serving. Admittedly the forces in Russia and on the Afghanistan borders must have a sufficiency of medical officers, as well as the Army of the Rhine and the troops in Great Britain. But all the same there is a strong suspicion entertained in well-informed quarters that many more temporarily commissioned medical officers could be released without the slightest detriment to anyone, if the Army Medical Service authorities and the War Office would take the trouble to put their house in order.

At present there are still married temporary medical officers detained abroad whose prospects of demobilisation are exceedingly vague. There are still also plenty of unmarried officers who were in private practice, and whose practices, of course, are steadily wasting away. This, admittedly, happened to thousands of medical men during the war, but it was then more or less inevitable, and everyone recognised that winning the war was of more importance than the financial troubles of individuals, serious though many of the latter were. But now the same compelling urgency does not hold good, and the A.M.S. authorities need once more to be made to recognise the duty they owe to the State and to their temporary officers. When a scandal arises concerning the medical arrangements for any campaign—*e.g.*, the Mesopotamia scandal, and on a lesser scale the recent Afghan business—the Army medical hierarchy always makes it an excuse for an increased establishment of medical officers, and that is doubtless what is happening just now. "Insufficient medical attention to the troops on the Frontier?" they reply to criticisms. "Oh! very well. Cancel those last sixty demobilisation orders for medical officers, and send them back to the Border." Whereas in truth it is not the number of medical officers on duty, but the way they are made use of, that makes the difference between efficiency and neglect. Throughout the war the Army Medical Service in France and other theatres of war was always overstocked with medical officers, a considerable percentage of whom could have been spared, even at the times of heaviest fighting (and a much higher proportion in slack times), if the organisation had been properly overseen. But the administrative heads could not be bothered to do this; it was so much simpler and easier to demand a few hundred more officers than to take trouble in economising and utilising to the best advantage those they had. And from what has lately been printed in the *Times*, the *Telegraph*, and *Truth*, it seems a certainty that this same vice of extravagance and carelessness is still hanging up the demobilisation of a lot of men who ought by this time to be back in civil practice. Meanwhile the country pays salaries which could be saved, the medical officers who receive them are kept idle, or comparatively idle, and there is a shortage of medical men at home which bears hardly on the public.

